# Increased concentrations of Serum amyloid A in dogs with sepsis caused by pyometra

**DOI:** 10.1186/s12917-014-0273-9

**Published:** 2014-11-28

**Authors:** Supranee Jitpean, Ann Pettersson, Odd V Höglund, Bodil Ström Holst, Ulf Olsson, Ragnvi Hagman

**Affiliations:** Department of Clinical Sciences, Swedish University of Agricultural Sciences, Box 7054, , SE-750 07 Uppsala, Sweden; Department of Surgery and Theriogenology, Faculty of Veterinary Medicine, Khon Kaen University, Khon Kaen, 40002 Thailand; Department of Economics, Applied Statistics and Mathematics, Swedish University of Agricultural Sciences, Box 7013, SE-750 07 Uppsala, Sweden

**Keywords:** SAA, CRP, SIRS, Bitch, Acute phase protein

## Abstract

**Background:**

Sepsis is a serious and potentially life-threatening condition and early diagnosis and appropriate treatment is crucial for survival. Pyometra is one of the most common diseases in intact female dogs. The disease often leads to sepsis (systemic inflammatory response syndrome, SIRS, caused by infection). Diagnostic markers for detecting sepsis are gaining increasing interest in veterinary medicine. Acute phase proteins (APPs) such as C-reactive protein (CRP) are useful for detecting systemic inflammation in dogs. Serum amyloid A (SAA) is another major APP in dogs that is not yet as widely used. Albumin is regarded as a negative APP and has earlier been evaluated for prediction of prognosis in septic dogs. The aim of the present study was to determine SAA, CRP and albumin concentrations in dogs with sepsis and pyometra and to evaluate whether these inflammatory markers are associated with length of postoperative hospitalization.

**Results:**

Thirty-one surgically treated bitches with pyometra were included, whereof 23 septic (SIRS-positive) and eight non-septic (SIRS-negative). Albumin concentrations were analyzed by routine automated methods. SAA and CRP analyses were performed with previously validated commercially available assays (ELISA and immunoturbidimetric).

Mean (±SE) serum concentrations of SAA were significantly higher in septic (130.8 ± 8.0 mg/L) compared to non-septic bitches (88.5 ± 12.5 mg/L). Using a cut-off value for SAA of 109.07 mg/L (n = 31 bitches), the sensitivity and specificity for detecting sepsis was 74% and 50%, respectively. Serum albumin concentrations were not significantly different in septic compared to non-septic bitches (mean ± SE, 25 ± 1 g/L and 26 ± 1 g/L, respectively). CRP concentrations were also not significantly different in septic (mean ± SE 225.6 ± 16.0 mg/L) compared to non-septic bitches (mean ± SE, 176.0 ± 27.1 mg/L). None of these inflammatory markers were associated with the outcome as measured by length of hospitalization.

**Conclusions:**

SAA concentrations were increased in dogs with sepsis induced by pyometra and may be useful as an adjunctive diagnostic marker for sepsis. To evaluate the full potential of SAA as a marker for sepsis also in other diseases, further studies are warranted.

## Background

Pyometra, purulent bacterial infection of the uterus, is a common diagnosis in intact female dogs. Differences in incidence rates between breeds are reported [1,2]. Ovariohysterectomy (OHE) is the treatment of choice, but is not always life-saving. The mortality ranges between 3-10% [2,3]. Gram-negative bacteria, mainly *Escherichia coli* (*E. coli*), are most often isolated from the uterus [4,5]. Gram-negative bacteria contain endotoxin which is released during bacterial growth or death, and endotoxin present in the bloodstream is a potent inducer of systemic inflammation [6]. Several studies have reported that the majority of dogs with pyometra also suffer from sepsis (*i.e.* sepsis defined as systemic inflammatory syndrome, SIRS, caused by infection) [7,8]. Sepsis is a serious condition frequently leading to organ dysfunctions in animals and humans. Despite modern treatments, sepsis associated with organ dysfunctions is a main cause of death (more than 40%) in human intensive care units [9]. Early diagnosis and appropriate treatment of sepsis is crucial for survival [10]. Diagnosing sepsis can be challenging because clinical signs and results of laboratory variable analyses are not unique for septic patients and there is no reliable diagnostic marker. In pyometra, certain clinical and laboratory analysis results can be valuable in the prediction of outcome as measured by prolonged postoperative hospitalization and presence of peritonitis [3]. The bacterial infection of the uterus stimulates the release of cytokines, lymphokines and other chemical mediators, which in turn trigger hepatic production of acute phase proteins [7,11,12]. Successful surgical treatment of the disease leads to rapid improvement of abnormalities in hematological, biochemical and inflammatory variables [3,13]. In human medicine, analysis of C-reactive protein (CRP) and procalcitonin are widely used for diagnosing inflammatory diseases and to predict outcome and increased concentrations are observed in septic patients [14-23]. In veterinary medicine, CRP and SAA have been investigated for diagnostic and prognostic purposes in several diseases [7,24-28]. Moreover, it has been reported that SAA analysis was valuable for identifying malignancy associated with inflammation in humans [29-32]. In dogs, it has been reported that SAA concentrations are less frequently increased in healthy dogs compared to CRP which indicates that SAA may be a more specific marker for systemic inflammation [33,34]. Albumin is considered to be a negative acute phase protein because serum concentrations decrease in inflammation and/or infection. Hypoalbuminemia in response to infection or inflammation is likely due to decreased production by the liver and/or increased vascular permeability that may lead to extravasal accumulation of albumin [35]. Albumin concentrations decrease in sepsis and may be valuable as a negative prognostic biomarker for survival [36-38]. Although controversial, albumin has been used as adjunctive treatment in septic animals [39,40]. Serum albumin is routinely analyzed in clinical laboratories and therefore accessible in most veterinary clinics. SAA has so far not been investigated in sepsis and associations between SAA, CRP and albumin have not been assessed in canine pyometra. The aim of the present study was to determine SAA, CRP and albumin concentrations in dogs with sepsis and pyometra and to evaluate whether these inflammatory markers are associated with length of postoperative hospitalization.

## Results

### Animals-general data

#### Physical examination findings

In this study, common clinical signs of pyometra included depression, anorexia, vaginal discharge, polydipsia, and polyuria (Table 1).

**Table 1:** **Case history and physical examination findings in 31 bitches with pyometra**

**Variable**	**Number of bitches with abnormality of variable/number of bitches with pyometra (n = 31)**
Case history	
Depression	31 (100%)
Moderate	30 (97%)
Severe	1 (3%)
Anorexia	23 (74%)
Vaginal discharge	20 (65%)
Polydipsia	14 (45%)
Polyuria	13 (42%)
Vomiting	4 (13%)
Diarrhea	0 (0%)
Physical examination	
Dehydration	29 (94%)
Fever	13 (42%)
Hypothermia	1 (3%)
CRT (CRT >2 sec)	2 (6%)
Mucous membranes	
Pale	2 (6%)
Hyperemic	4 (13%)
Abdominal pain on palpation	3 (7%)

CRT = Capillary refill time.

#### Bacteriological findings

*Escherichia coli (E. coli)* was isolated from the uterine content in 25/31 bitches (81%). Other bacterial species isolated were β-hemolytic streptococci in 7/31 bitches (23%) and *Proteus spp* in 1/31 bitch (3%). Of these, two bitches had growth of two bacterial species, one bitch with *E. coli* and β-hemolytic streptococci, one bitch with *Proteus spp* and β-hemolytic streptococci.

Bacterial blood cultures were positive in three of 27 sampled bitches (11%) and *E. coli* was demonstrated in two bitches and β-hemolytic streptococci in one bitch (the same pathogens were also demonstrated in the uterine cultures from each of these bitches).

### Sepsis

In all bitches with pyometra, the mean age and weight was 7.7 ± 2.4 years and 27.1 ± 11.7 kg. The mean age and weight in the septic group was 8.1 ± 2.3 years and 28.9 ± 12.5 kg. In the non-septic group the mean age and weight was 6.6 ± 2.6 years and 21.9 ± 7.5 kg. The weight and age did not differ between the septic and non-septic bitches.

#### Laboratory variables

The number of band neutrophils, monocytes and basophils were significantly higher in septic bitches compared to the non-septic group (p <0.05) (Table 2).

**Table 2:** **Clinical and laboratory findings in the 23 septic and 8 non-septic bitches with pyometra**

	**Pyometra**			
**Variable**	**Septic mean ± SE (n) (range)**	**Non-septic mean ± SE (n) (range)**	**p value (ANOVA)**	**Reference range** ^**†**^
BT (°C)	39.2 ± 0.1 (23) (38.3-40.5)	38.6 ± 0.2 (8) (37.8-39.4)	0.005	38-39^¶^
RR (breaths per minute)	69 ± 14 (17) (15-260)	19 ± 22 (7) (16-32)	0.08	20-40^¶^
HR (beats per minute)	106 ± 4 (20) (72-150)	94 ± 9 (8) (80-110)	0.15	80-120^¶^
Hemoglobin (g/L)	132 ± 4 (23) (93-173)	116 ± 7 (8) (89-134)	0.06	132-199^†^
Hematocrit (%)	37 ± 1 (28-40)	34 ± 2 (25-36)	0.2	38-57^†^
WBC (×10^9^/L)	24.2 ± 3.0 (23) (2.8-106.4)	16.7 ± 5.0 (8) (11.3-31.2)	0.2	5.8-16.0^†^
Neutrophils (×10^9^/L)	13.9 ± 2.6 (23) (1.4-85.1)	11.8 ± 4.4 (8) (7.1-22.5)	0.7	3.0-11.5^†^
Band neutrophils (×10^9^/L)	5.5 ± 0.7 (23) (0.6-4.4)	2.1 ± 1.3 (8) (0.6-18.1)	0.02	0.0-0.3^†^
Lymphocytes (×10^9^/L)	1.9 ± 0.2 (23) (0-4.9)	1.2 ± 0.4 (8) (0.2-2.4)	0.17	1.4-4.8^†^
Monocyte (×10^9^/L)	2.6 ± 0.3 (23) (0.2-6.4)	1.4 ± 0.4 (8) (0.2-4.4)	0.03	0.2-1.4^†^
Eosinophils (×10^9^/L)	0.3 ± 0.1 (23) (0-1.9)	0.2 ± 0.2 (8) (0-0.6)	0.7	0.1-1.2^†^
Basophils (×10^9^/L)	0 ± 0 (23) (0-0)	0.08 ± 0.03 (8) (0-0.6)	0.03	0.0-0.1^†^
Bile acids (μmol/L)	4.5 ± 1.4 (23) (0.3-20.4)	4.9 ± 2.4 (8) (0.3-12.3)	0.9	0.0-12^†^
ALT (μkat/L)	0.4 ± 0.1 (22) (0.05-1.3)	0.4 ± 0.1 (8) (0.2-0.7)	0.9	0.0-1.3^†^
Glucose (mmol/L)	4.9 ± 0.2 (20) (2.9-6.8)	5.4 ± 0.4 (7) (2.9-7.3)	0.3	4.5-5.8^†^
BUN (mmol/L)	3.6 ± 0.3 (23) (1.5-7.4)	2.8 ± 0.5 (8) (1.6-3.6)	0.3	2.5-8.8^†^
Serum creatinine (μmol/L)	66 ± 3 (22) (48-100)	61 ± 5 (8) (41-81)	0.4	40-130^†^

BT = Body temperature, RR = Respiratory rate, HR = Heart rate, WBC = Total white blood cell count, ALT = Alanine aminotransferase, BUN = Blood urea nitrogen.

^†^Reference range (at the Clinical Pathology Laboratory, University Animal Hospital, Swedish University for Agricultural Sciences, Uppsala).

^¶^Reference range by Ettinger and Feldman, Textbook of Veterinary Internal Medicine, 2010 [41].

#### Inflammatory markers

**Figure 1:**
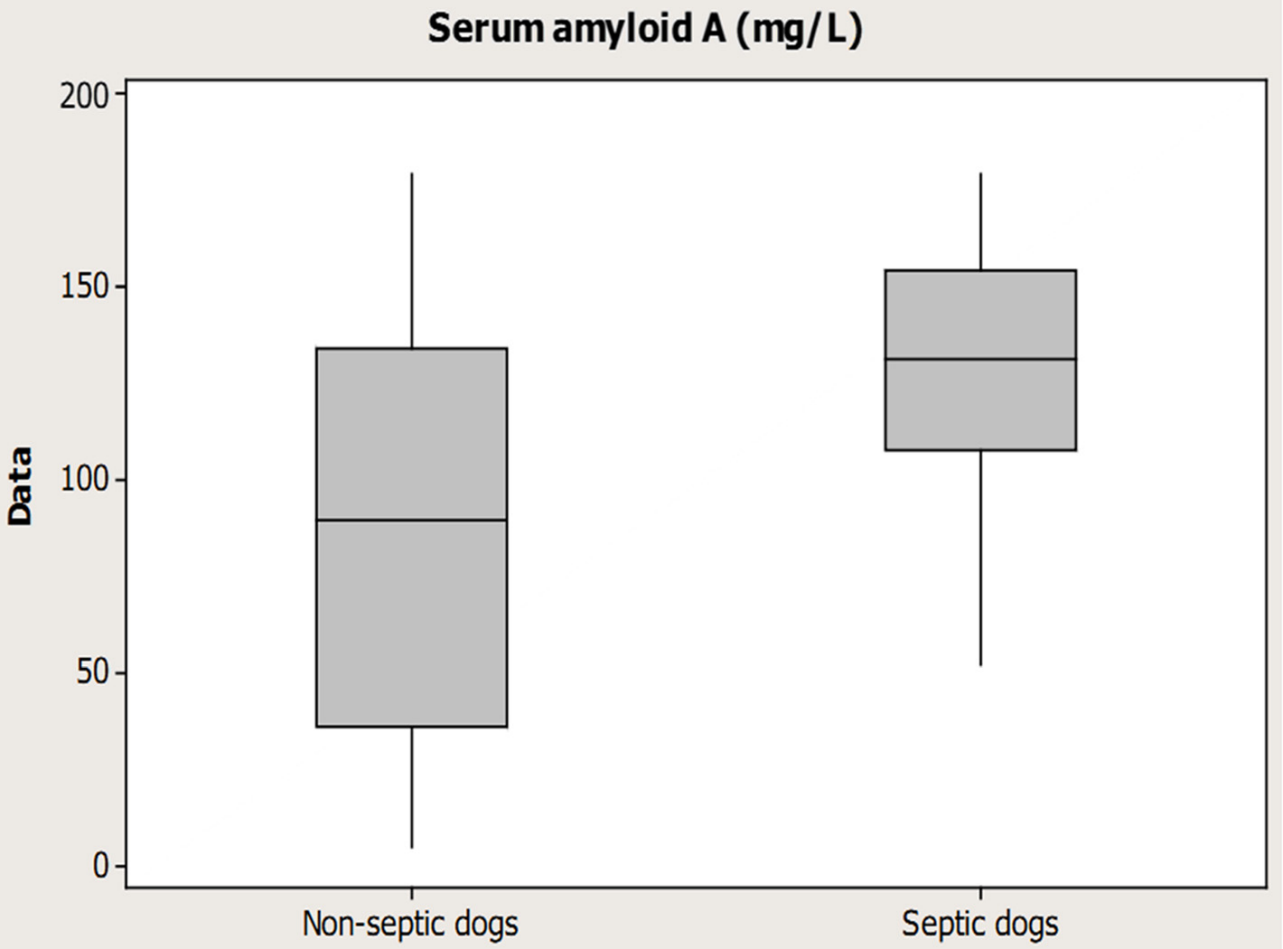
**Boxplot graph illustrating Serum amyloid A concentrations in bitches with pyometra.** Eight non-septic bitches and 23 septic dogs with pyometra including in this study.

**Figure 2:**
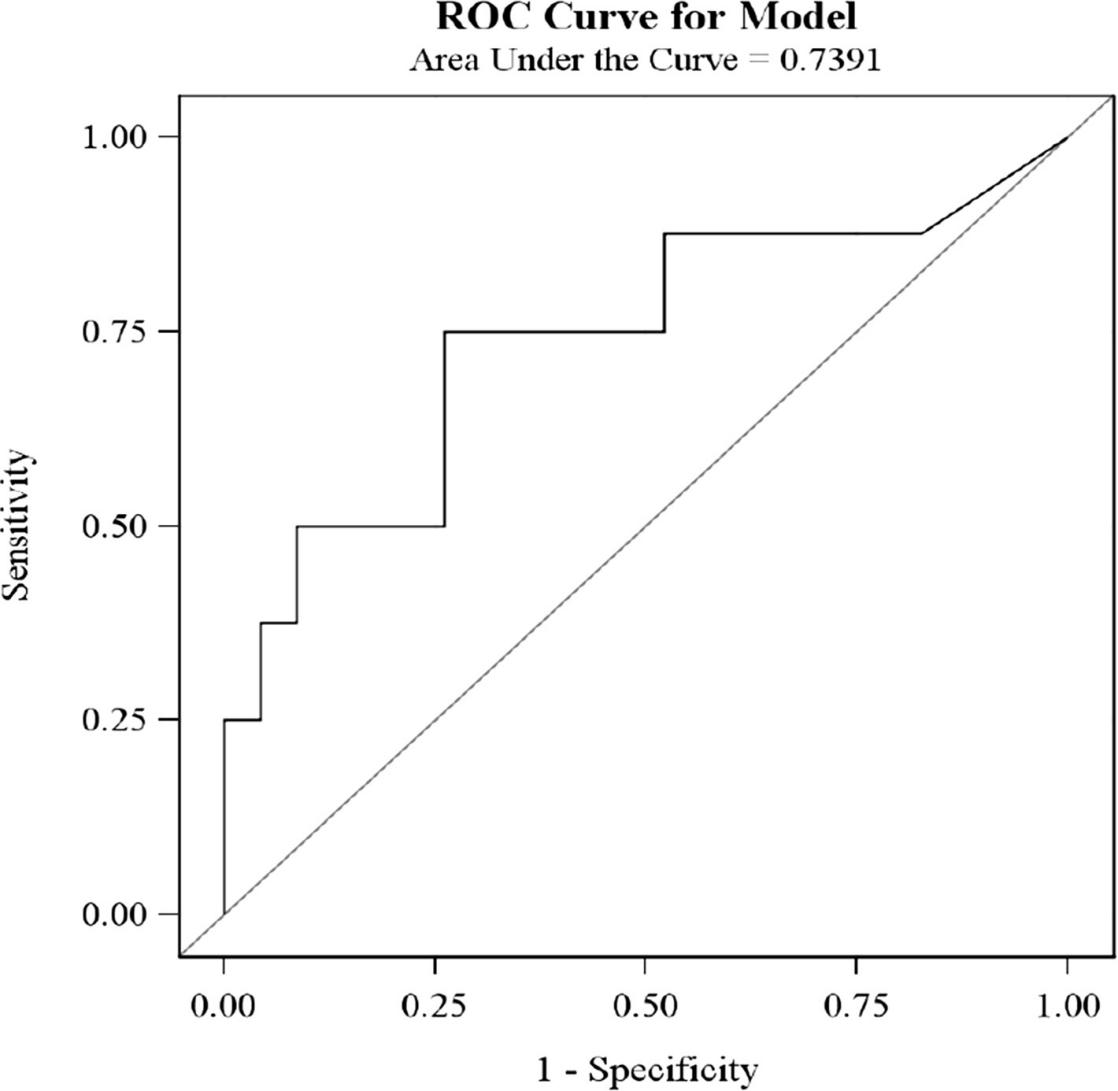
**Receiver operating curve (ROC) illustrating the diagnostic power of Serum amyloid A (SAA).** There were investigated in 31 bitches with pyometra.

The mean SAA concentration were significantly higher in septic compared to non-septic bitches (p = 0.006) (Table 3 and Figure 1). If a cut-off value of SAA of 109.07 mg/L was selected (n = 31 bitches), the sensitivity and specificity for detecting sepsis was 74% and 50%, respectively. The Receiver operating characteristic curve (ROC) analysis displayed an area under the curve (AUC) of 0.74 for SAA in the dogs with sepsis (p = 0.04) (Figure 2). In four bitches with sepsis and one non-septic bitch, the SAA concentration exceeded 180 mg/L (sample dilution up to 1/8 000). The mean concentrations of CRP and serum albumin were not significantly higher in septic compared to non-septic bitches (p = 0.1 and p = 0.7, respectively) (Table 3). In the three bitches with positive blood cultures (bacteremia), the concentrations of CRP were 97, 272 and 298 mg/L, concentrations of SAA were 56.6, 114.7 and 117.9 mg/L and concentrations of albumin 21, 26 and 20 g/L. Four out of 23 bitches in septic group required prolonged hospitalization. In the non-septic group, none of the bitches stayed longer in the hospital than two days after surgery. None of the inflammatory markers investigated (SAA, CRP, albumin) were associated with prolonged postoperative hospitalization.

**Table 3:** **Mean ± SE serum concentrations of Serum amyloid A (SAA), C-reactive protein (CRP) and albumin in septic and non-septic bitches with pyometra**

	**Pyometra (n = 31)**		
**Variable**	**Septic mean ± SE (n = 23) (range)**	**Non-septic mean ± SE (n = 8) (range)**	**p value (ANOVA)**
SAA (mg/L)	130.8 ± 8.0 (52.3-180.0)	88.5 ± 12.5 (5.0-180.0)	0.006
CRP (mg/L)	225.6 ± 16.0 (49.0-362.0)	176.0 ± 27.1 (2.5-316.0)	0.1
Serum albumin (g/L)	25 ± 1 (16-32)	26 ± 1 (21-33)	0.7

## Discussion

Concentrations of SAA were higher in bitches with sepsis compared to those without sepsis. This is the first time that a possible clinical value of SAA analysis for identification of sepsis in dogs is reported. However, the cut-off value for SAA of 109.07 mg/L used, yielded a sensitivity of 74% for detecting bitches with sepsis which is lower than the 97% sensitivity reported previously when using the clinical criteria HR, RR, WBC/percentage band neutrophils and rectal temperature for determination of SIRS [42]. Using criteria for sepsis that have high sensitivity is more important than a high specificity because including non-septic bitches in the septic group would not endanger their condition. But to include truly septic bitches in the non-septic group with less optimal treatment could have serious consequences for the individual bitch. A specificity of 50% means that SAA cannot be used as a single variable for diagnosing sepsis because 50% truly septic bitches would be classified as non-septic. The low specificity was not surprising because SAA is regarded as an unspecific variable because concentrations increase in all diseases with systemic inflammation. However, SAA could be a potential adjunctive marker for sepsis in dogs, if used in combination with the clinical SIRS criteria or other markers that increase the sensitivity and specificity for sepsis diagnosis. SAA concentrations were significantly increased in septic dogs which points to a possible higher clinical value for SAA in the detection of sepsis compared with CRP, and this potential deserves to be further investigated. A larger study is necessary to investigate if a clinically useful cut-off value for SAA in sepsis (with higher sensitivity and specificity) can be established.

In this study, the finding of a clinical value of SAA is in line with other studies in which SAA has shown more promise as a diagnostic marker than CRP for gastrointestinal disorders linked with inflammation [33,34]. In human medicine, SAA has also been shown to be a better prognostic marker for severity of cancer associated with inflammation [29,30,32,43]. Though not caused by infection, this points to a diverse capacity of CRP and SAA and that there are conditions or diseases for which SAA could have a clinically higher diagnostic or prognostic value than the more frequently used CRP. That CRP and SAA levels have different diagnostic abilities in spite of both being major acute phase proteins in dogs is in agreement with the findings of one study in which CRP concentrations were only moderately positively correlated with SAA concentrations [44].

Concentrations of albumin were not significantly different in bitches with or without sepsis. This finding was unforeseen since lower albumin concentrations have been reported in sepsis and decrease in experimentally induced endotoxemia in dogs, cats and rats [35,45,46]. However, the clinical situation is different from experimental studies in that the studied dogs were admitted at various stages of disease progression and the effect of endotoxemia may therefore be less prominent. Albumin might have a diagnostic value in dogs with pyometra and more severe disease (septic peritonitis or septic shock), but this needs to be further studied. Hypoalbuminemia was earlier detected in 81% (25 out of 31, data not shown) of these bitches with pyometra which is in line with other reports and also indicate that albumin could be interesting to study as a possible sepsis marker [35,45,46]. Gram-negative bacteria, foremost *E. coli,* were isolated from the uterine content which is similar to most other studies of the disease [4,5,47]. That pyometra induces hypoalbuminemia is not surprising because hypoalbuminemia has been reported as an effect of Gram-negative infection and bacteremia [45]. To date, no study has specifically investigated albumin as a marker for sepsis in dogs. The results presented here suggest that albumin could be clinically useful as an adjunctive marker for diagnosis of pyometra, but albumin does not seem to be as valuable as SAA in the detection of sepsis.

Increased concentrations of CRP have previously been shown to be associated with presence of SIRS and prolonged hospitalization in bitches with pyometra, supporting the usefulness of CRP analysis in clinical practice [7]. Moreover, increased CRP concentrations have been associated with increased mortality in dogs with SIRS and sepsis [26]. In the present study, higher CRP concentrations were not associated with sepsis which is in contrast to a previous report [7]. This report was larger, including 30 septic and 23 non-septic bitches, which could somewhat explain the difference in results, since otherwise the sampling methods and patient inclusion criteria were similar. Pyometra often induces sepsis, as demonstrated in the present study where 74% of the bitches were SIRS-positive. This proportion is of the same magnitude (57-69%) as has been reported previously in the disease [7,47]. However, by using the selected SIRS-criteria with a specificity of 64%, 36% non-septic bitches will be included in the septic group [42]. There are several criteria are available to define SIRS in dogs, based on the different cut-off values with different specificity and sensitivity [42,48,49]. Using SIRS-criteria with the highest sensitivity (97%) available is advisable since it is important to not clinically misdiagnose a truly septic dog as non-septic which could lead to less optimal monitoring and treatment resulting in a poor outcome. However, the lack of more specific clinical criteria available for sepsis is a limitation of the study. Another limitation is that the samples were freeze-stored up to 8 months before analysis. However, CRP and SAA have been shown to be very stable at room temperature and storage [50-53]. Depression, anorexia, polydipsia/polyuria and vaginal discharge are common clinical signs in bitches with pyometra, emphasizing the systemic effects of the illness in the studied bitches [3,25,47]. Of all hematology and biochemistry variables analyzed, band neutrophils, basophils and monocytes were increased in septic bitches. These results were in line of other studies in response to infection and chronic disease [54,55]. Because band neutrophils and WBC are included in the criteria used to define sepsis, these and other variables linked with WBC can therefore not be considered as independent. For prediction outcome, as measured by length of hospitalization, none of the markers analyzed in the present study were useful, but a larger study material would be beneficial for further evaluation.

## Conclusion

SAA concentrations were increased in dogs with sepsis induced by pyometra whereas concentrations of CRP and albumin did not differ between septic and non-septic bitches. This indicates that SAA may have a potential clinical value for the detection of sepsis. The selected cut-off value to investigate the ability of SAA as marker for sepsis resulted in relatively low sensitivity and specificity suggesting that SAA cannot be used as a single marker for this purpose. Further studies of larger patient groups should be performed to evaluate the potential value of SAA in the detection of sepsis in dogs.

## Methods

### Study design and ethical approval

The study was designed as a prospective clinical study and was approved by the Uppsala Local Ethical Board (permission number C413/12). A signed informed consent was obtained from the dog-owner before participation of their dog in the study.

### Animals

Thirty-one client-owned bitches of 18 different breeds, Airedale Terrier ( n = 1), Beagle (n = 1), Bernese Mountain Dog (n = 2), Cane corso (n = 1), Cavalier King Charles Spaniel (n = 2), Chow-Chow (n = 1), Collie (n = 2), Drever (Swedish hound) (n = 1), English Springer Spaniel (n = 1), German Shepherd Dog (n = 3), Giant Schnauzer (n = 2), Golden Retriever (n = 3), Labrador Retriever (n = 1), Mixed-Breed Dog (n = 3), Münsterländer (n = 1), Newfoundland (n = 1), Staffordshire Bull Terrier (n = 4) and Standard Poodle (n = 1) were included in the study performed as part of a project concerning inflammation [44]. Complete physical examination was performed by the veterinarian in charge, and the results filled in a special form. Preliminary diagnosis of pyometra was based on case history data, finding on physical examination and diagnostic imaging by either abdominal ultrasonography or radiology or both. All bitches were treated by OHE at the University Animal Hospital (UDS), Swedish University of Agricultural Sciences (SLU), Uppsala, during 2011. The bitches were selected based on owner agreement, daytime admission (Clinical Pathology Laboratory access) and that at least one of the authors was on clinical rotation. The diagnosis was confirmed by postoperative macroscopic identification of a pus-filled uterus, positive bacterial culture from the uterine content and histopathological examination of formaldehyde-fixated uteri and ovaries (inclusion criteria as previously described for the pyometra group included in a study published earlier). Bitches with the histopathological diagnosis mucometra, hydrometra or cystic endometrial hyperplasia were excluded [44].

The admitting clinician completed a form specifying body temperature (BT), heart rate (HR), respiratory rate (RR), mucus membrane color, capillary refilling time (CRT), pain response at abdominal palpation, hydration status and general attitude at the time of admission [44].

In general, bitches that are surgically treated for pyometra are hospitalized at UDS for 1–2 days after surgery. Only bitches with specific complications or poor general condition stay longer in the animal hospital and ≥3 days hospitalization was therefore defined as prolonged.

### Sepsis

Sepsis was defined as presence of SIRS caused by infection (pyometra). Presence of SIRS was determined according to definitions and criteria by Hauptman and others (1997) [42], with fulfilment of two or more of the following criteria: (1) Body temperature (BT) < 38.1°C (100.4°F) or > 39.2°C (102.6°F); (2) Heart rate (HR) > 120 beats per min; (3) Respiratory rate (RR) > 20 breaths per minute; and Total white blood cell count (WBC) < 6 or > 16 × 10^3^/μL, or percentage band neutrophils (PBN) > 3%.

### Bacterial culturing

Samples were immediately collected from the content of the removed uterus with sterile fiber cotton swabs (Culturette; Becton-Dickinson AG, Stockholm, Sweden). Bacterial cultures and sensitivity tests were performed at the accredited laboratory, Section of Bacteriology, National Veterinary Institute (SVA), Uppsala, Sweden as earlier described [56].

### Blood sampling and laboratory tests

#### Hematological and, biochemical analyses

Prior to surgery, blood samples for hematology and biochemistry were aseptically collected from the distal cephalic vein and transferred into EDTA and non-additive collection tubes (Vacutainer®, Becton-Dickinson, Stockholm, Sweden). The non-additive tubes were centrifuged and serum separated before analysis of biochemistry parameters. Hematological (WBC including differential counts, hematocrit (EVF) and hemoglobin (Hb)) and biochemical (Bile acids, Alanine aminotransferase (ALT), glucose Blood urea nitrogen (BUN), and Creatinine) were performed (Advia 2120; Siemens Healthcare Diagnostics, Deer-field, IL, USA for hematology and Abbott Architect c4000, Abbott Park, IL, USA, for biochemistry). Albumin was analyzed with a colorimetric method (bromocresol green) using an automated analyzer (Abbott Architect c4000, Abbott Park, IL, USA) with a commercial albumin reagent from Abbott Laboratories. All laboratory analyses were performed according to the routine methods at the accredited Clinical pathology laboratory, UDS, SLU, Uppsala, Sweden. After centrifugation, all serum not used for biochemistry analyses was directly transferred in aliquots of 200 μl to cryogenic vials (NuncCryoTubes, VWR International, Stockholm, Sweden), and freeze-stored at −80°C until analysis of SAA and CRP, up to eight months before analysis. Blood samples for bacterial cultures were aseptically collected into a sterile syringe and 3 ml blood was added to two biphasic aerobic and anaerobic medium blood culture bottles (BOF, Substratlab, SVA, Uppsala, Sweden). The BOFs were transported to the accredited laboratory, Section of Bacteriology, SVA, Uppsala, Sweden, cultured in 37°C for 7 days or until growth and bacterial strains subsequently typed.

#### Analysis of acute phase proteins

Analysis of SAA was performed by trained laboratory staff at the Clinical Pathology Laboratory, UDS, with a commercially available ELISA (Tridelta Phase^™^ Range SAA Assay, Tridelta Development Limited, County Kildare, Ireland), with mean intra- and inter-assay coefficients of variation of 4.75% and 8.8%, respectively, and with the lowest measurable concentration of 10 mg/L. The absorbance was evaluated using Tecan Sunrise reader (Tecan Inc., Männedorf, Switzerland). The method has previously been validated for dogs [49]. A human immunoturbidimetric CRP assay that has been validated for dogs, was used to measure serum CRP concentrations [50]. The analyses were performed on Abbot Architect (Abbott Architect c4000, Abbott Park, IL, USA) and the method was calibrated with canine CRP (Life Diagnostics canine CRP, West Chester, USA). The lowest measurable concentration was 5 mg/L with a mean intra- and interassay variation were 1.4% and 2.4%. Samples with high concentrations of CRP (above 217 and 225 mg/L for the two lots used) were autodiluted 1:3 with 0.9% NaCl and reanalyzed to obtain exact values.

### Statistical analyses

All statistical analyses were performed by the use of SAS 9.3 package (SAS Institute Inc. Cary, NC, USA). ANOVA was used to evaluate the differences in SAA, CRP and albumin results between septic and non-septic groups and to investigate possible relations between inflammatory markers and morbidity as measured by increased postoperative hospitalization. Residual diagnostic plots were used to assess normality and homoscedasticity.

The diagnostic power of different markers was assessed using logistic regression [57]. The area under curve (AUC) was used as a measure of diagnostic ability. The cut-off value was selected as upper 5% limit in the non-septic group [58].

The significance level was set to p < 0.05 for all tests used in the study.

Descriptive data was described as mean ± SE. Bitches with concentrations below the lowest measurable concentration were assigned a value of half that value for the statistical analyses.

## References

[1] Jitpean S, Hagman R, Holst BS, Hoglund OV, Pettersson A, Egenvall A (2012). Breed variations in the incidence of pyometra and mammary tumours in Swedish dogs. Reprod Domest Anim.

[2] Egenvall A, Hagman R, Bonnett BN, Hedhammar A, Olson P, Lagerstedt AS (2001). Breed risk of pyometra in insured dogs in Sweden. J Vet Intern Med.

[3] Jitpean S, Strom-Holst B, Emanuelson U, Hoglund OV, Pettersson A, Alneryd-Bull C, Hagman R (2014). Outcome of pyometra in female dogs and predictors of peritonitis and prolonged postoperative hospitalization in surgically treated cases. BMC Vet Res.

[4] Hagman R, Greko C (2005). Antimicrobial resistance in Escherichia coli isolated from bitches with pyometra and from urine samples from other dogs. Vet Rec.

[5] Vandeplassche M, Coryn M, Deschepper J (1991). Pyometra in the bitch- cytological, bacterial, histological and endocrinologic characteristics. Vlaams Diergen Tijds.

[6] Vandeventer SJH, Buller HR, Tencate JW, Sturk A, Pauw W (1988). Endotoxaemia- an early predictor of septicemia in febrile patients. Lancet.

[7] Fransson BA, Lagerstedt AS, Bergstrom A, Hagman R, Park JS, Chew BP, Evans MA, Ragle CA (2007). C-reactive protein, tumor necrosis factor alpha, and interleukin-6 in dogs with pyometra and SIRS. J Vet Emerg Crit Car.

[8] Hagman R, Reezigt BJ, Ledin HB, Karlstam E (2009). Blood lactate levels in 31 female dogs with pyometra. Acta Vet Scand.

[9] Vincent J-L, Nelson DR, Williams MD (2011). Is worsening multiple organ failure the cause of death in patients with severe sepsis?. Crit Care Med.

[10] Kumar A, Roberts D, Wood KE, Light B, Parrillo JE, Sharma S, Suppes R, Feinstein D, Zanotti S, Taiberg L, Gurka D, Cheang M (2006). Duration of hypotension before initiation of effective antimicrobial therapy is the critical determinant of survival in human septic shock. Crit Care Med.

[11] Dabrowski R, Kocki T, Szczubial M, Dabrowski W, Parada-Turska J (2013). Kynurenic acid in plasma and endometrium in bitches with pyometra. Inflammation.

[12] Karlsson I, Wernersson S, Ambrosen A, Kindahl H, Sodersten F, Wang L, Hagman R (2013). Increased concentrations of C-reactive protein but not high-mobility group box 1 in dogs with naturally occurring sepsis. Vet Immunol Immunopathol.

[13] Bartoskova A, Vitasek R, Leva L, Faldyna M (2007). Hysterectomy leads to fast improvement of haematological and immunological parameters in bitches with pyometra. J Small Anim Pract.

[14] Pierrakos C, Vincent J-L (2010). Sepsis biomarkers: a review. Crit Care.

[15] Jebali MA, Hausfater P, Abbes Z, Aouni Z, Riou B, Ferjani M (2007). Assessment of the accuracy of procalcitonin to diagnose postoperative infection after cardiac surgery. Anesthesiology.

[16] Prat C, Manuel Sancho J, Dominguez J, Xicoy B, Gimenez M, Ferra C, Blanco S, Lacoma A, Maria Ribera J, Ausina V (2008). Evaluation of procalcitonin, neopterin, C-reactive protein, IL-6 and IL-8 as a diagnostic marker of infection in patients with febrile neutropenia. Leuk Lymphoma.

[17] Van Nieuwkoop C, Bonten TN, van’t Wout JW, Kuijper EJ, Groeneveld GH, Becker MJ, Koster T, Wattel-Louis GH, Delfos NM, Ablij HC, Leyten EMS, Van Dissel JT (2010). Procalcitonin reflects bacteremia and bacterial load in urosepsis syndrome: a prospective observational study. Crit Care.

[18] Wagle S, Grauaug A, Kohan R, Evans SF (1994). C-reactive protein as a diagnostic-tool of sepsis in very immature babies. J Paediatr Child Health.

[19] Berger C, Uehlinger J, Ghelfi D, Blau N, Fanconi S (1995). Comparison of C-reactive protein and white blood cell count with differential in neonates at risk for septicemia. Eur J Pediatr.

[20] Meisner M, Tschaikowsky K, Beier W, Schuttler J: **Procalcitonin (PCT) - A novel parameter for diagnosis and monitoring of bacterial inflammation and sepsis.** *Anasth Intensivmed* 1996, **37**(10):529–&.

[21] Oczenski W, Fitzgerald RD, Schwarz S (1998). Procalcitonin: a new parameter for the diagnosis of bacterial infection in the peri-operative period. Eur J Anaesth.

[22] Guven H, Altintop L, Baydin A, Esen S, Aygun D, Hokelek M, Doganay Z, Bek Y (2002). Diagnostic value of procalcitonin levels as an early indicator of sepsis. Am J Emerg Med.

[23] Claeys R, Vinken S, Spapen H, Elst KV, Decochez K, Huyghens L, Gorus FK (2002). Plasma procalcitonin and C-reactive protein in acute septic shock: clinical and biological correlates. Crit Care Med.

[24] Dabrowski R, Wawron W, Kostro K (2007). Changes in CRP, SAA and haptoglobin produced in response to ovariohysterectomy in healthy bitches and those with pyometra. Theriogenology.

[25] Fransson BA, Karlstam E, Bergstrom A, Lagerstedt AS, Park JS, Evans MA, Ragle CA (2004). C-reactive protein in the differentiation of pyometra from cystic endometrial hyperplasia/mucometra in dogs. J Am Anim Hosp Assoc.

[26] Gebhardt C, Hirschberger J, Rau S, Arndt G, Krainer K, Schweigert FJ, Brunnberg L, Kaspers B, Kohn B (2009). Use of C-reactive protein to predict outcome in dogs with systemic inflammatory response syndrome or sepsis. J Vet Emerg Crit Car.

[27] Griebsch C, Arndt G, Raila J, Schweigert FJ, Kohn B (2009). C-reactive protein concentration in dogs with primary immune-mediated hemolytic anemia. Vet Clin Path.

[28] Nakamura M, Takahashi M, Ohno K, Koshino A, Nakashima K, Setoguchi A, Fujlno Y, Tsujimoto H (2008). C-reactive protein concentration in dogs with various diseases. J Vet Med Sci.

[29] Zhang G, Sun X, Lv H, Yang X, Kang X (2012). Serum amyloid A: a new potential serum marker correlated with the stage of breast cancer. Oncol Lett.

[30] Wang J-Y, Zheng Y-Z, Yang J, Lin Y-H, Dai S-Q, Zhang G, Liu W-L (2012). Elevated levels of serum amyloid A indicate poor prognosis in patients with esophageal squamous cell carcinoma. BMC Cancer.

[31] Ramankulov A, Lein M, Johannsen M, Schrader M, Miller K, Loening SA, Jung K (2008). Serum amyloid A as indicator of distant metastases but not as early tumor marker in patients with renal cell carcinoma. Cancer Lett.

[32] Cho WCS, Yip TT, Cheng WW, Au JSK (2010). Serum amyloid A is elevated in the serum of lung cancer patients with poor prognosis. Br J Cancer.

[33] Christensen BM, Langhorn R, Goddard A, Andreasen BE, Moldal E, Tvarijonaviciute A, Kirpensteijn J, Jakobsen S, Persson F, Kjelgaard-Hansen M (2013). Canine serum amyloid A (SAA) measured by automated latex agglutination turbidimetry is useful for routine sensitive and specific detection of systemic inflammation in a general clinical setting. J Vet Med Sci.

[34] Christensen BM, Langhorn R, Goddard A, Andreasen BE, Tvarijonaviciute A, Kirpensteijn J, Jakobsen S, Persson F, Kjelgaard-Hansen M (2014). Comparison of serum amyloid A and C-reactive protein as diagnostic markers of systemic inflammation in dogs. Can Vet J.

[35] Deysine M, Stein S (1980). Albumin shifts across the extracellular-space secondary to experimental infections. Surg Gynecol Obstet.

[36] Ralphs SC, Jessen CR, Lipowitz AJ (2003). Risk factors for leakage following intestinal anastomosis in dogs and cats: 115 cases (1991–2000). J Am Vet Med Assoc.

[37] Bentley AM, Otto CM, Shofer FS (2007). Comparison of dogs with septic peritonitis: 1988–1993 versus 1999–2003. J Vet Emerg Crit Car.

[38] Goldwasser P, Feldman J (1997). Association of serum albumin and mortality risk. J Clin Epidemiol.

[39] Mazzaferro EM, Rudloff E, Kirby R (2002). The role of albumin replacement in the critically ill veterinary patient. J Vet Emerg Crit Car.

[40] Craft EM, Powell LL (2012). The use of canine-specific albumin in dogs with septic peritonitis. J Vet Emerg Crit Car.

[41] Ettinger SJ, Feldman E (2010). Textbook of Vetrinary Internal Medicine.

[42] Hauptman JG, Walshaw R, Olivier NB (1997). Evaluation of the sensitivity and specificity of diagnostic criteria for sepsis in dogs. Vet Surg.

[43] Kosuge M, Ebina T, Ishikawa T, Hibi K, Tsukahara K, Okuda J, Iwahashi N, Ozaki H, Yano H, Kusama K, Nakati T, Umemura S, Kimura K (2007). Serum amyloid A is a better predictor of clinical outcomes than C-reactive protein in non-ST-segment elevation acute coronary syndromes. Circ J.

[44] Jitpean S, Holst BS, Hoglund OV, Pettersson A, Olsson U, Strage E, Sodersten F, Hagman R (2014). Serum insulin-like growth factor-I, iron, C-reactive protein, and serum amyloid A for prediction of outcome in dogs with pyometra. Theriogenology.

[45] Greiner M, Wolf G, Hartmann K (2008). A retrospective study of the clinical presentation of 140 dogs and 39 cats with bacteraemia. J Small Anim Pract.

[46] Powanda MC, Wannemac Rw, Cockerel Gl: **Nitrogen-metabolism and protein-synthesis during pneumococcal sepsis in rats.** *Infect Immun* 1972, **6**(3):266–&. 10.1128/iai.6.3.266-271.1972 PMC422526 4404682

[47] Hagman R (2011). Serum alpha-1-acid glycoprotein concentrations in 26 dogs with pyometra. Vet Clin Path.

[48] Otto CM (2007). Clinical trials in spontaneous disease in dogs: a new paradigm for investigations of sepsis. J Vet Emerg Crit Car.

[49] Okano S, Yoshida M, Fukushima U, Higuchi S, Takase K, Hagio M (2002). Usefulness of systemic inflammatory response syndrome criteria as an index for prognosis judgement. Vet Rec.

[50] Hillstrom A, Hagman R, Tvedten H, Kjelgaard-Hansen M (2014). Validation of a commercially available automated canine-specific immunoturbidimetric method for measuring canine C-reactive protein. Vet Clin Pathol.

[51] Hillstrom A, Tvedten H, Lilliehook I (2010). Evaluation of an in-clinic Serum Amyloid A (SAA) assay and assessment of the effects of storage on SAA samples. Acta Vet Scand.

[52] Christensen M, Jacobsen S, Ichiyanagi T, Kjelgaard-Hansen M (2012). Evaluation of an automated assay based on monoclonal anti-human serum amyloid A (SAA) antibodies for measurement of canine, feline, and equine SAA. Vet J.

[53] Kjelgaard-Hansen M (2004). Canine C-Reactive Protein. PhD Thesis.

[54] Fransson B, Lagerstedt AS, Hellmen E, Jonsson P (1997). Bacteriological findings, blood chemistry profile and plasma endotoxin levels in bitches with pyometra or other uterine diseases. J Vet Med A.

[55] Wheaton LG, Johnson AL, Parker AJ, Kneller SK (1989). Results and complications of surgical-treatment of pyometra- a review of 80 cases. J Am Anim Hosp Assoc.

[56] Hagman R, Karlstam E, Persson S, Kindahl H (2009). Plasma PGF(2 alpha) metabolite levels in cats with uterine disease. Theriogenology.

[57] Olsson U (2002). Generalized Linear Models – an Applied Approach.

[58] Pape MS (2003). The Statistical Evaluation of Medicine Tests for Classification and Prediction.

